# Case report: A rare secondary systemic candidiasis as a bite wound complication in a dog

**DOI:** 10.3389/fvets.2024.1418194

**Published:** 2024-10-23

**Authors:** Yun-Joo Geum, Hyun-Jung Han

**Affiliations:** Department of Veterinary Emergency and Critical Care, College of Veterinary Medicine, Konkuk University, Seoul, Republic of Korea

**Keywords:** antifungal agent, itraconazole, bite wound, *Candida*, systemic candidiasis

## Abstract

An 11-year-old, 4.8 kg, intact male mixed-breed dog was evaluated for a bite wound that had occurred a day prior to consultation. On examination, the patient exhibited signs of early to-late decompensatory shock, hemothorax, pneumothorax, and rib fractures. Initial shock management and resuscitation were performed. After several days of stabilization, exploratory thoracotomy, thoracic wall reconstruction, culture sampling, and antibiotic susceptibility tests were conducted. Empirical antimicrobial treatments were performed while pending culture results. Despite aggressive antimicrobial therapy, the patient had focal seizures and wound dehiscence, presumably due to the worsening of infection and inflammation. Necrotic tissues adjacent to the dehiscence were debrided, and the wound was opened. A previous analysis of wound and blood cultures identified *Candida glabrata*, and itraconazole was initiated in accordance with the culture results. Successful treatment was achieved, and the wound was closed. The patient remained healthy after 2 months of monitoring. To the best of our knowledge, this was the first case report of systemic candidiasis in a dog secondary to a bite wound diagnosed via blood culture. Additionally, this case highlights successful treatment with itraconazole.

## 1 Introduction

*Candida* is a type of yeast belonging to the family *Saccharomycetaceae* (1). *Candida* normally inhabits the mammalian mucous membranes in the gastrointestinal, genital, upper respiratory tracts, and oral cavity. Immunosuppressed patients can obtain this opportunistic infection through breaks in the mucous membrane barrier, which causes candidiasis (2). Common risk factors for *Candida* infections include diabetes mellitus, use of broad-spectrum antimicrobials or corticosteroids, intravenous (IV) catheter placement, and provision of parenteral nutrition (1). Candidiasis encompasses all *Candida*-related infections affecting cutaneous, mucosal, blood, and deep-seated organs (2). *Candida* infections in the blood, referred to as candidemia, and those penetrating deep-seated organs are defined as invasive candidiasis (2).

In humans, *Candida* is the most common fungal infective agent (3). Many *Candida* spp. can cause candidiasis; however approximately 90% of candidiasis cases are caused by the following five species, listed in descending order: *Candida albicans*, *Candida glabrata*, *Candida tropicalis*, *Candida parapsilosis*, *and Candida krusei* (3). Although *C. albicans* is the most common cause of candidiasis, recently there has been a significant increase in non-albicans *Candida* spp. infections due to fluconazole resistance (4). *Candida* can disseminate through both superficial and systemic routes (4).

Similar to humans, *Candida* can opportunistically infect immunosuppressed animals (5), such as those with parvovirus infection (6). There are various candidiasis cases wherein *Candida* is a naturally occurring fungi including cutaneous candidiasis (7), *Candida-*related urinary tract infection (8–10), and intestinal candidiasis (11). In dogs, candidiasis can occur not only as an opportunistic infection in the mucous membrane but also in several other locations such as the peritoneum (12, 13), bone (14), joints (15), and eyes (16). However, there are only a few reports of systemic candidiasis including pyogranulomatous lymphadenitis (17, 18) and candidemia (19). Several studies have reported cases of *Candida* infection in animals; however *C. glabrata* infection in dogs is rare (13, 14). Although there have been cases of *Candida* infection in humans bitten by dogs (20, 21), there have been no reported cases of *Candida* infection in dogs bitten by other dogs. Here, we report the diagnosis and successful management of a *Candida* infection secondary to a bite wound in a dog.

## 2 Case description

**Figure 1:**
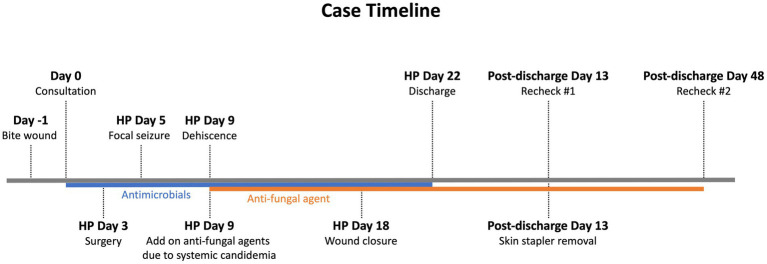
Case timeline. The case description and treatment are summarized in this timeline. Antimicrobials were prescribed empirically upon consultation. However, on day 5 of hospitalization, the patient experienced a focal seizure. On day 9 of hospitalization, *C. glabrata* was cultured from the blood, indicating systemic candidemia, thus an anti-fungal agent, itraconazole, was administered. After itraconazole treatment, the wound successfully granulated. Wound closure was conducted on day 18 of hospitalization. The patient was discharged 4 days after the wound closure without any complications. Recheck monitoring was performed on days 13 and 48 post-discharge. HP, hospitalization.

An 11-year-old male mixed-breed dog weighing 4.8 kg presented to the Department of Emergency and Critical Care at the Konkuk University Veterinary Medical Teaching Hospital. The day before the consultation, the dog was bitten by another dog (Jindo breed) (Figure 1). Emergency stabilization and suturing of the herniated intestine near the left inguinal region were performed at the local animal hospital. The wound sites included the abdomen, thorax, and near the vertebrae. The patient was referred to our hospital due to respiratory instability and suspected pulmonary damage secondary to multiple rib fractures.

**Figure 2:**
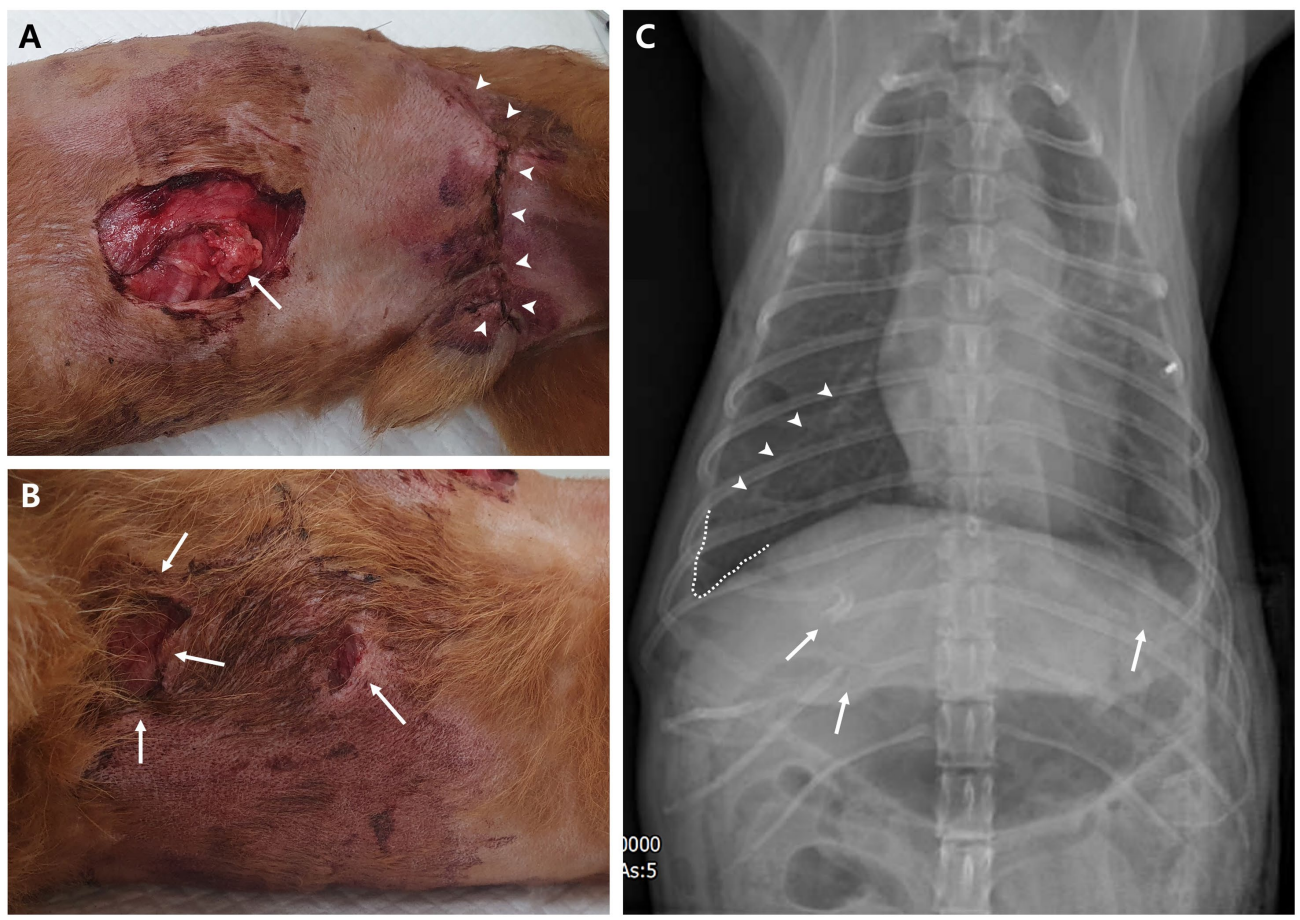
Physical examination and diagnostic image at presentation. **(A)** Ventral left thoracic open wound with herniated soft tissue (arrow) and left inguinal wound (arrowheads), which had been closed at the local animal hospital; observed in the right lateral recumbent position. **(B)** Ventral right thoracic open wounds (arrows) near the sternum observed in the same position. **(C)** Dorsoventral thoracic radiography reveals fractures in the right 11th–12th rib and left 8th rib (arrows). A fissure line (arrowheads) between the right middle and caudal lobe of the lung and blunting of the right costophrenic sulcus indicate lung retraction due to pneumothorax or pleural effusion, such as hemothorax. In addition, overall lung opacity is elevated.

Upon presentation, the patient was diagnosed with early to-late decompensatory shock and suspected septic shock. Physical examination revealed a rectal temperature of 34.6°C, heart rate of 160/min, respiratory rate of 44/min, and systolic blood pressure of 50 mmHg with weak pulse. The mucous membrane was pale pink, and the capillary refill time was slightly delayed at 2 s. Additionally, the dog exhibited signs of 5% dehydration. There was also a large open wound on the left ventral thoracic region exposing the subcutaneous layer and herniated tissue, along with a left inguinal wound that had been closed at the local animal hospital (Figures 2A,B). The patient also had a seizure episode and was administered diazepam (0.5 mg/kg IV).

Crystalloid at 1.5 times the shock dose (30 mL/kg) and turbostarch at 0.5 times the shock dose (2.5 mL/kg) were provided to address the hypotension. However, the systolic blood pressure did not improve; thus, norepinephrine was initiated at a constant rate infusion of 0.1 μg/kg/min and later increased to 0.2 μg/kg/min. Systolic blood pressure eventually returned to normotensive range (100–140 mmHg), although the dog remained stuporous to semi-comatose. A serum biochemical profile showed increased levels of C-reactive protein (9.1 mg/dL; reference interval, 0.1–1 mg/dL), lactate (5.41 mmol/L; reference interval, 0.5–2.5 mmol/L), glucose (149 mg/dL; reference interval, 70–143 mg/dL), blood urea nitrogen (85 mg/dL, reference interval, 7–27 mg/dL), creatinine (3.4 mg/dL, reference interval, 0.5–1.8 mg/dL), phosphorus (14.3 mg/dL, reference interval, 2.5–6.8 mg/dL), and d-dimer (1947.81 ng/mL, reference interval, 50–250 ng/mL), and hypercoagulable state in thromboelastography. Right lateral and dorsoventral thoracic radiographs revealed concurrent fractures of the 8th left and 11th–12th right ribs, along with pneumothorax and hemothorax (Figure 2C). Additional ultrasonography performed the next day demonstrated atelectasis of the right lung, indicating pulmonary contusion.

**Figure 3:**
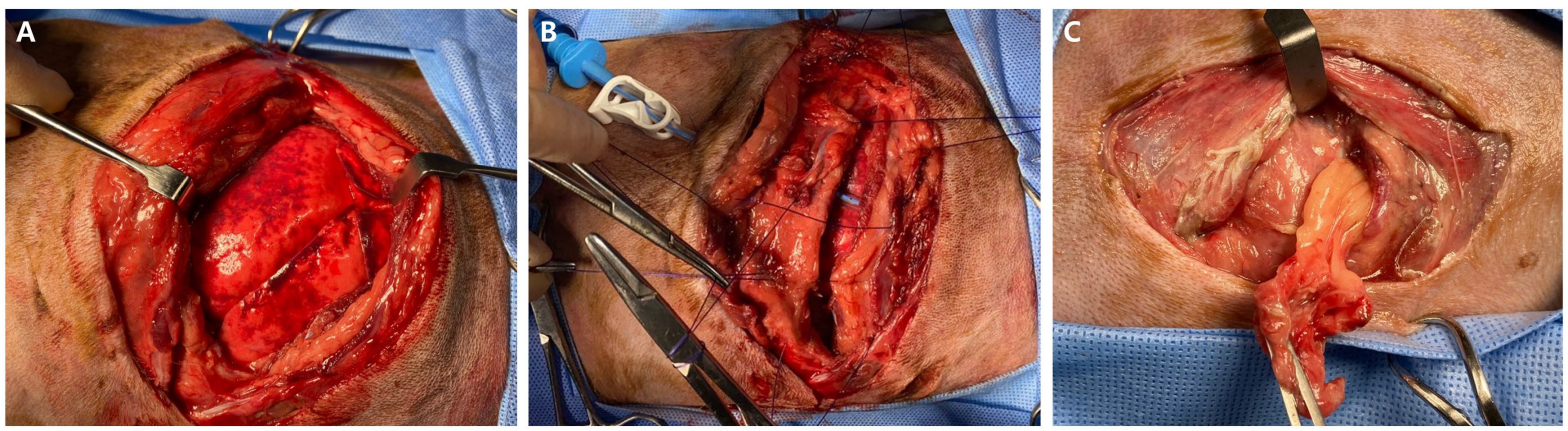
Macroscopic images during surgery. **(A)** Intrathoracic exploration of the right thoracic cavity is conducted revealing significant pulmonary contusion on the right middle and caudal lobe. **(B)** Serosanguinous pleural fluid and necrotic tissue are observed around the fracture of right 11th rib, and lung swab samples were collected for aerobic and anaerobic microorganism cultures and fungal cultures. Following the samplings, the intrathoracic cavity and thoracic wall were lavaged and closed routinely. Before the routine thoracic closure, a 12 Fr chest tube was inserted with a trocar through the right 8th intercostal space. **(C)** Left intrathoracic exploration revealed that the herniated tissue at the left thoracic open wound is abdominal fat.

The thoracic wounds were managed as open wounds with cleansing and bandaging for 3 days. Subsequently, the patient stabilized, and no additional necrosis or discharge was detected from the wounds. On day 3 of hospitalization, surgery was performed under general anesthesia to reconstruct the thoracic wall, and to debride and close the bite wound. Anesthesia was induced with midazolam (3 mg/kg, IV) and propofol (4 mg/kg, IV) and maintained with isoflurane in 100% O_2_. To explore the thoracic cavity and reconstruct the right thoracic wall, a right intercostal thoracotomy and closure were performed. The dog was placed in the left lateral recumbent position, and an incision was made in the right 8th intercostal space. Exploration of the thoracic cavity revealed intrathoracic serosanguinous fluid and significant pulmonary contusions in the right middle and caudal lobes (Figure 3A). Several samples were collected for aerobic and anaerobic microorganism and fungal cultures, including pleural effusion, necrotic tissue around the fracture of the right 11th rib, and lung swabs. Histopathological examination for necrotic tissue wasn’t conducted due to financial constraints of the owner. Following sampling, the intrathoracic cavity and thoracic wall were lavaged with warm saline, and reconstruction of the thoracic wall between the right 11th–13th ribs was performed. Routine thoracic closure was followed by appositioning several preplaced sutures around the right 11th rib adjacent to the incision and by suturing the serratus ventralis, scalenus, and pectoralis muscles with a simple continuous suture (22). Prior to routine thoracic closure, a 12 Fr chest tube (chest tube with sharp trocar, 12 Fr, 19 cm; Mila International, Florence, KY, USA) was inserted with a trocar through the right 8th intercostal space (Figure 3B). The subcutaneous tissues and skin were routinely closed.

After routine skin closure of the right thorax, the dog was repositioned in the right lateral recumbent for debridement and closure of the large open left ventral thoracic wound. The wound was lavaged with 0.9% normal saline and 0.05% chlorhexidine. Following removal of the hernial soft tissue, which was confirmed as abdominal fat (Figure 3C), standard wound closure was performed. Postoperatively, blood collected from the jugular vein was subjected to culture tests to diagnose systemic infections.

Postoperative supportive care included fluid therapy with isotonic crystalloids (Plasma Solution A), analgesia, aggressive antimicrobials, antiemetics, gastrointestinal protectants, and anticoagulant therapy. Analgesia was initially provided with fentanyl (4 μg/kg/h, IV) and lidocaine (1.2 mg/kg/h, IV) at constant rate infusion. On postoperative day 1, the combination constant rate infusion of fentanyl and lidocaine was discontinued and replaced with a fentanyl patch (4 μg/kg/h, TD) and meloxicam (0.1 mg/kg, PO, q24h). Empirical antimicrobial therapy including ceftazidime (20 mg/kg IV, q8h), metronidazole (15 mg/kg IV, q12h), and enrofloxacin (20 mg/kg IV, q24h) was initially administered. Based on the results of the antimicrobial susceptibility test, the antimicrobials were switched to amoxicillin/clavulanate (13.75 mg/kg, IV, q12h) and metronidazole (15 mg/kg, IV, q12h) to cover the etiologic agent identified, *Enterococcus faecalis,* although fungal culture results were still pending during this time. Maropitant (1 mg/kg, SC) and anticoagulants, including clopidogrel (loading dose: 10 mg/kg, PO, q24h; maintenance dose: 4 mg/kg, PO, q24h) and dalteparin (150 U/kg, SC, q8h), were initiated as well.

**Figure 4:**
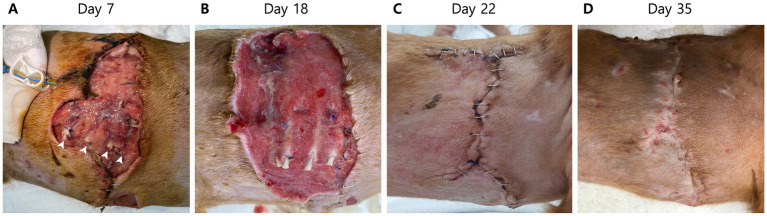
Macroscopic images of the right thoracic wound on days 7, 18, 22, and 35. **(A)** Dehiscence at the right thoracic surgical closure site due to ongoing infection and inflammation is observed on day 7 of hospitalization. Surgical debridement under mild sedation is performed. After the debridement, costochondral separation (arrowheads) is detected between the right 7th and 11th costochondral junction. **(B)** After administration of itraconazole, an antifungal agent, granulation tissue successfully forms for wound closure on day 18 of hospitalization. **(C)** Four days after the routine wound closure of the right thorax (day 22 of hospitalization), the wound is healing well without any inflammation or dehiscence. **(D)** Skin staples are removed 18 days after wound closure (35 days after the initial presentation) after adequate wound healing.

On day 5 of hospitalization, the patient experienced clonic facial twitching, a type of focal seizure. On day 9, dehiscence was observed at the right thoracic surgical site (Figure 4A). Additional dehiscence sampling was initiated for culture tests. Hematologic evaluation revealed moderate anemia with a red cell count of 3.87 × 10^12^/L (reference interval, 5.7–8.8 × 10^12^/L) and leukocytosis (53.25 × 10^9^/L; reference interval, 5–17 × 10^9^/L) along with concurrent lymphocytosis (36.62 × 10^3^/μL; reference interval, 1–5 × 10^3^/μL) and monocytosis (15.83 × 10^3^/μL; reference interval, 0.2–1.1 × 10^3^/μL). Several toxic neutrophils were detected on blood smear slides. A serum biochemical profile still showed an increased C-reactive protein level (6 mg/dL; reference interval, 0.1–1 mg/dL); although it was lower compared to that at the time of surgery. Therefore, there was still ongoing infection and inflammation. On day 9 of hospitalization, necrosis of the right thoracic wall worsened; therefore, debridement was performed under mild sedation. During the procedure, costochondral separation due to necrosis of the cartilage was detected between the right 7th and 10th costochondral junction (Figure 4A). After debridement, the open thoracic wound was managed with Manuka honey therapy to promote granulation tissue formation and reduce inflammation until wound closure.

All culture samples submitted to Nosvet laboratories (Gyeonggi-do, Republic of Korea) and were grown on blood agar, brucella, and sabouraud dextrose agar with chloramphenicol plate. If colonies were observed, species identification was performed using matrix assisted laser desorption ionization-time of flight mass spectrometry. After identification, antibiotic susceptibility test was conducted under Clinical and Laboratory Standards Institute 2018 guideline using the disc diffusion method.

No fungus was cultured from the pleural effusion sample. However, *C. glabrata* was cultured from the necrotic tissue around the right 11th rib fracture and lung swab samples. Furthermore, *C. glabrata* was cultured from the blood, indicating systemic candidemia. Therefore, itraconazole (10 mg/kg, PO, q24h) for *Candida* infection was added on day 9 of hospitalization.

After antifungal treatment, the patient’s level of consciousness improved, and the pleural effusion gradually decreased. On day 17 of hospitalization, the chest tube was removed as there was no longer pleural effusion. A blood examination on day 15 of hospitalization showed C-reactive protein level within the reference range (0.8 mg/dL; reference interval, 0.1–1 mg/dL). Although white blood cell counts (42.91 × 10^3^/μL; reference interval, 1–5 × 10^3^/μL) and D-dimer levels (1,226 ng/mL; reference interval, 50–250 ng/mL) were still above the reference intervals, they were steadily decreasing. On day 18 of hospitalization, the open thoracic wound produced sufficient granulation tissue for closure (Figure 4B); therefore, routine wound closure was performed. The patient was discharged 4 days after wound closure without any complications (Figure 4C).

The patient remained healthy as noted at follow-up visits 13 and 48 days post discharge (Figure 4D). Blood culture tests confirmed the absence of microorganisms and fungal growth. Thus, management was concluded. Additional oral swab samples from the bitten dog and samples from the soil where the patient lived were collected to identify the infection route of *Candida*. From the oral culture, *Candida guilliermondii*, now revised as *Meyerozyma guilliermondii*, and other microorganisms were identified, whereas in the soil culture no *Candida* spp. or other fungi were identified.

## 3 Discussion

To the best of our knowledge, this is the first documented case of systemic candidiasis secondary to a bite wound in a dog. Because it was a fungal systemic infection, empirical antimicrobial treatment was ineffective, and the patient exhibited focal seizures and wound dehiscence. After the blood culture results identified *C. glabrata*, the antifungal agent itraconazole was initiated with noted patient improvement.

Systemic fungal infections mostly occur through the invasion of a wound site or direct inhalation into the lungs. For example, *C. albicans* can cause systemic fungal infections through medical devices such as IV catheters (23). Although *C. albicans* is a resident fungi in the skin or mucosal layer, it can infect patients in immunosuppressive conditions or through direct invasion (23), such as from a bite wound as in the current case. There are numerous organisms in the oral cavity, and they can enter the injury site through biting, leading to polymicrobial infection (24). One study, using ribosomal DNA genomic analysis, identified *C. albicans* as the second most dominant species cultured from oral swab samples of healthy domestic dogs, after *Malassezia pachydermatis*. *C. glabrata, C. auris*, and *C. parapsilosis* were also isolated (25).

The pathogenesis of *C. glabrata* infection differs from that of *C. albicans* infection. Unlike *C. albicans*, *C. glabrata* cannot actively penetrate the epithelium in the absence of hyphae. Instead, it relies on endocytosis and not only survives but also replicates inside macrophages (26). In humans, two major causative species of oropharyngeal candidiasis are *C. albicans* and *C. glabrata*, and they usually co-infect because *C. glabrata* alone is non-invasive to the oropharyngeal mucous epithelium, in contrast to its invasiveness in gastric mucous epithelium (27). The adherence of *C. glabrata* to the hyphae of *C. albicans* worsens the severity of oropharyngeal candidiasis (27). Therefore, *C. glabrata* in the oral mucous epithelium of the bitten dog is non-invasive; it can present as a commensal organism. However, *C. glabrata* in this patient was considered to have invaded the thoracic cavity through the bite wound, which subsequently spread to the systemic circulation and led to systemic candidiasis. In a mouse model, *C. glabrata* changed its growth type after adapting to macrophages, enabling it to escape phagocytes and increase its virulence (28).

Regarding the bite wound, the most probable source of infection is the oral microbiota of the biting animal (24). Less commonly, pathogens may originate from environmental sources or the patient’s skin (24). To identify the infection route of *C. glabrata*, oral swab samples from the biting dog and soil samples from the area where the patient lived were collected. From the oral sample, *C. guilliermondii* (now reclassified as *Meyerozyma guilliermondii*) and other microorganisms including *Enterobacter cloacae*, *Aspergillus niger*, and *Rhodotorula mucilaginosa* were cultured. No *Candida* spp. were found in the soil culture, although *Fusarium oxysporum* and *Neopestalotiopsis* were identified. The culture test performed on the wound at the time of presentation identified *Enterococcus faecalis*, a microorganism not typically found in the skin’s normal flora (29).

Dog bite occurred the day before presentation, and septic shock was strongly suspected upon arrival. Therefore, the likelihood of nosocomial infection, such as from a kennel, causing sepsis is very low. Additionally, *C. glabrata* was detected in multiple samples collected during surgery and in blood cultures, the possibility of sampling error is also low.

The different culture results between the biting dog and the patient can be attributed to several reasons. First, there was the possibility of *Candida* mutation. During prolonged *Candida* infections in immunocompromised patients, more than one *Candida* species and strain can coexist in a single host. For example, a retrospective study of fungal urinary tract infections in dogs and cats revealed multiple *Candida* infections, such as *C. albicans*, *C. tropicalis*, and *C. krusei* (30). In addition, *Candida* strains and phenotypes can be altered through genetic switching. The pathogenesis of the white-opaque phenotypic switching of *C. albicans* is well known in *Candida* spp. (31). Second, there was a difference in the timing of the cultures. An oral swab from the biting dog was obtained 3 months later, allowing sufficient time for potential changes in oral microbial distribution. There are some reports on the shift in oral microbiota over 1 month due to dietary supplements, including dental chews (32) and age-related change (33).

We acknowledge the limitation that the culture test conducted 3 months later from the bitten dog may have a few association with the *Candida* found in the bite wound patient. However, the presence of *Candida* suggests the possibility of a secondary systemic infection as a bite wound complication. Therefore, in cases of severe bite wounds, conducting oral swab cultures from the biting animal may be recommended to use appropriate antimicrobials and prevent sepsis.

Empirical antifungal agents are not commonly used to treat bite wounds because systemic fungal infections due to bite wounds have only been reported in a few cases, although there has been a human case treated with an antifungal agent after a cat bite wound that did not improve with antimicrobial treatment (34). However, such cases are relatively rare. Moreover, in human medicine, empirical antimicrobials are not commonly prescribed to treat dog bite wounds without suspicion of bacterial infections (35). In veterinary medicine, nearly 80% of cases are treated with prophylactic antimicrobials (36, 37). Although antimicrobials are commonly used in veterinary medicine, antifungal agents are not. Accordingly, the current patient was initially prescribed an aggressive antimicrobial treatment, and no antifungal agents were prescribed until the fungal culture results were determined. Unfortunately, the fungal culture required a longer incubation time than the bacterial culture owing to slow growth (38), thus delaying the results and leading to wound deterioration due to prolonged inflammation despite aggressive antimicrobial treatment and wound management. Therefore, fungal culture is essential. Given that it takes a long time to confirm fungal culture results, empirical antifungal treatment should be considered even before the fungal culture is confirmed in bite wound patients who have a poor response to antimicrobial treatment.

Treatments for candidiasis in humans and animals are similar. In humans, the first-line medicine is an echinocandin followed by fluconazole and lipid formulation amphotericin B (39). *C. albicans* typically responds well to azoles, but *C. glabrata* may require higher doses of fluconazole due to resistance to azoles (39). Fluconazole is the preferred first-line treatment in veterinary medicine for several reasons including a low occurrence of adverse effects, minimal hepatic metabolism, extensive renal elimination, and cost effectiveness (40, 41). However, clinical trials for vaginal candidiasis in humans have shown that itraconazole is more effective than fluconazole (42). Moreover, the occurrence rate of invasive fungal infections, including *Candida* spp., was relatively lower with prophylactic use of itraconazole than with fluconazole (43). Although the costs differ, itraconazole and fluconazole exhibit similar therapeutic effects, reoccurrence rate, and hepatotoxicity for blastomycosis treatment in dogs (44). There have been a few cases where itraconazole was used to treat candidiasis (14, 45). In this patient, owing to its effectiveness, itraconazole was administered as the first-line treatment and resulted in a positive response. Further research is needed due to the limited number of cases involving itraconazole treatment. This report describes the effective use of itraconazole for the treatment of candidiasis.

## 4 Conclusion

Systemic candidiasis resulting from a bite wound, as reported in this study, is the first documented case in dogs. When managing bite wounds, it is essential to consider the possibility of fungal infections and to conduct fungal culture tests initially. If infection and inflammation persist despite aggressive antimicrobial treatment, the administration of empirical antifungal agents such as itraconazole should be considered.

## Data Availability

The original contributions presented in the study are included in the article/supplementary material, further inquiries can be directed to the corresponding author.
